# Metabolic Disease, NAD Metabolism, Nicotinamide Riboside, and the Gut Microbiome: Connecting the Dots from the Gut to Physiology

**DOI:** 10.1128/msystems.01223-21

**Published:** 2022-01-25

**Authors:** Anthony A. Sauve

**Affiliations:** a Department of Pharmacology, Weill Cornell Medical College, New York, New York, USA

**Keywords:** NAD^+^, NAD^+^ metabolism, nicotinamide riboside, energy metabolism, metabolic syndrome, microbiome

## Abstract

The effort to use nutrients as interventions to treat human disease has been important to medicine. A current example in this vein pertains to NAD^+^ boosters, such as nicotinamide riboside (NR) and nicotinamide mononucleotide (NMN), which are in many clinical trials in a variety of disease conditions. Independent laboratories have shown that ingested NR (or NMN) has mitigating effects on metabolic syndrome in mice. V. V. Lozada-Fernández, O. deLeon, S. L. Kellogg, F. L. Saravia, et al. (mSystems 7:e00230-21, 2022, https://doi.org/10.1128/mSystems.00230-21) show that NR shifts gut microbiome contents and that the transplantation of an NR-conditioned microbiome by fecal transfer reproduces some effects of NR in mice on a high-fat diet. The involvement of the gut microbiome as a factor in NR effects is linked to changes to the gut microbiome and its activity to transform NR and downstream catabolites. This commentary draws attention to these findings and focuses on some puzzling aspects of NAD^+^ boosters, exploring the still murky interactions between NAD^+^ metabolism, energy homeostasis, and the gut microbiome.

## COMMENTARY

The gut microbiome is a key player in energy and metabolic homeostasis in humans and has dynamic effects that are still being appreciated. One aspect of the microbiome’s contribution to human health and well-being is its interaction with dietary intake, leading to physiological effects such as the alteration of the chemical structure of ingested food components and the modulation of human metabolism in the body (1). Dietary intake can also change the compositions and/or metabolic activities of the microbiome (1–3). How these effects might be involved in the actions of ingested nutritional compositions is a topic of heightened interest. These issues are addressed head-on in a recent publication by Lozada-Fernández et al. (4), where they consider the effects of the NAD^+^ precursor nicotinamide riboside (NR) on the gut microbiome and investigate the contributions of the microbiome to the observed mitigating effects of NR in a mouse model of metabolic syndrome.

NR is a metabolic precursor to NAD^+^ and is metabolized in the gut to nicotinamide and in the gut microbiome to nicotinic acid (5). The ingestion of NR provides enhanced concentrations of NAD^+^ precursors and bioactive NAD^+^-related metabolites in the gastrointestinal (GI) lumen. NR has drawn increased attention as a 21st-century NAD^+^ precursor, stimulated by the identification and characterization of two human kinases, Nrk1 and Nrk2 (6). The kinases have been shown to phosphorylate NR to nicotinamide mononucleotide (NMN), which is a direct precursor to NAD^+^ (7). Work done by the Sauve laboratory over 15 years ago showed that NR has special NAD^+^-enhancing characteristics in mammalian cells (8). Subsequent work, by Auwerx and colleagues, showed that NR provided as an oral pharmacophore could prevent weight gain and improve insulin sensitivity in mice treated with a high-fat diet (HFD) (9). These findings with NR administration have been reproduced by other researchers, and metabolic improvements have been observed in other mouse models of metabolic syndrome (10, 11).

Nevertheless, the effects of NR have been puzzling in some respects. In mice, oral NR is believed to break down in the gastrointestinal lumen prior to participation in nonintestinal systemic NAD^+^ metabolism as shown by isotope-labeling studies (12). Thus, at least in mice, the majority of the *in vivo* effects of NR have not been linked to NR passing intact into the bloodstream but rather have been linked to prior conversion to downstream metabolites such as nicotinamide (12) or possibly nicotinic acid (5). Interestingly, elevated appearances of nicotinic acid adenine dinucleotide in mouse tissues are clearly associated with NR administration (13). These downstream nicotinate-related metabolites would be predicted to arise from activities not encoded by the host genome but rather contributed by the gut microbiome, such as via the microbe enzymes NMN deamidase and/or nicotinamidase (5). These enzymes can convert nicotinamide-containing metabolites to nicotinic acid-containing metabolites. The putative involvement of the microbiome in NR actions strengthens consideration that the gut microbiome contributes to NR effects in metabolic syndrome.

Lozada-Fernández et al. (4) provide an intriguing investigation of this question. They ask if NR alters metabolism in the gut microbiome of mice during HFD treatment. Furthermore, they ask if NR alters the composition of the microbiome. As expected, NR addition to diet blunts excess weight gain and improves glucose homeostasis in this model, as noted by other authors (9). Meanwhile, NR increases the fecal release of short-chain fatty acids such as propionate, butyrate, valerate, and isobutyrate and also shifts the composition of the gut microbiome to *Firmicutes*, which are butyrate producers in the GI lumen. Although interesting, this shift does not require the conclusion that microbiome changes provide NR-induced mitigating effects in HFD-fed mice. Importantly, shifts in the gut microbiome content induced by NR have also been observed by other researchers (14, 15). Previous studies have used fecal material transfer (FMT) with NR treatment but in the context of alcohol-induced changes in behavior. The application of FMT to address the effects of NR on metabolic syndrome is a key forward step for this study.

**FIG 1:**
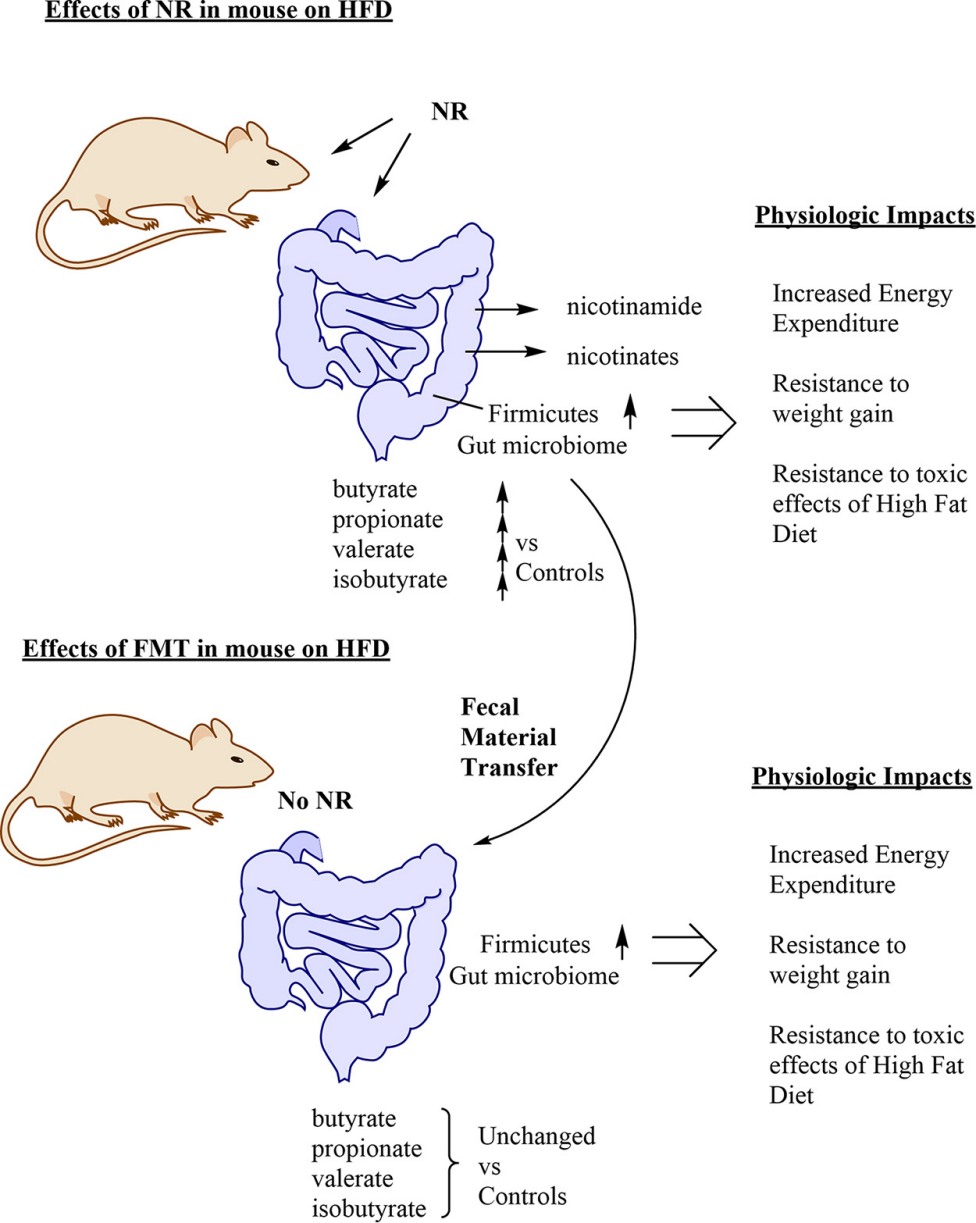
Depiction of the fates and consequences of ingested nicotinamide riboside (NR) in mice and the effects of fecal material transfer (FMT). NR is broken down in the gastrointestinal lumen and produces products such as nicotinamide, which is passed in the bloodstream. NR induces a shift in the microbiome to cosymbionts called *Firmicutes*. These microbes produce short-chain fatty acids such as propionate, butyrate, isobutyrate, and valerate, which are detected in feces in increased abundances versus controls. The consequences of the use of NR in high-fat diet (HFD)-treated mice include resistance to weight gain, improvements in glucose regulation, and improvements in insulin sensitivity. In the FMT experiments described in the text, NR-conditioned transfer to naive mice causes resistance to weight gain and energy expenditures in recipient mice fed an HFD. Interestingly, fecal outputs for short-chain fatty acids are not significantly increased in these mice. The implications for these experiments are discussed in the text.

To address the possibility that the observed microbiome shift is functional, Lozada-Fernández et al. utilized FMT to determine if NR-induced changes in the microbiome contribute to resistance to weight gain in HFD-fed mice. Strikingly, they find that the transfer of the NR-conditioned microbiome, which is high in gut butyrate producers, can provide metabolic benefits otherwise linked to NR. They transferred these microbiomes to naive mice, also receiving HFD but not receiving NR (Fig. 1). The transfer provided resistance to weight gain. It might be the case that these transfers cause this effect for unidentified reasons not linked to NR, but some of the observed effects are similar. For example, NR-conditioned fecal transfer causes increased energy expenditure, similar to the effect of NR intervention (Fig. 1).

The wide availability of NR and the abundance of clinical trials of NR in human subjects for conditions as diverse as Alzheimer’s disease, metabolic syndrome, and heart conditions and even for the treatment of coronavirus disease 2019 (COVID-19) infection/symptoms make it of increased relevance to understand how this compound might be altering human physiology (16–18). The observation that NR can alter the gut microbiota in mice highlights the complexity and impact of this nutrient/supplement and raises further questions about its use in humans. For example, what are the human microbiome-altering effects of NR in humans, and do these contribute to NR effects in people? An important concern is that recent clinical studies have reported weak effects of NR dosing in people with mild obesity or prediabetic conditions (18, 19), somewhat inconsistent with the strong effects on metabolism observed in mice. Exceptionally, though, recent work with oral NMN found significant insulin-sensitizing effects in prediabetic women (20). Is it the case that the effects of NR seen in mice do not translate well to humans because the effects on the human microbiome are distinctly different from those on mice, or is the dosing in humans simply insufficient? How to rationalize the differences observed for experimental outcomes with the use of NR and NMN has also been unexplained.

These new studies by no means provide the last word on how pharmacological manipulation of NAD^+^ metabolism can impact systemic energy metabolism and actually highlight the complexity of the problem. For example, the mechanisms for how an NR-conditioned microbiome might mediate resistance to the toxic effects of an HFD are not convincingly elucidated in this study. It still seems unclear how microbiome-linked increased production of short-chain fatty acids can have such effects. Does an NR-conditioned microbiome have special properties that involve augmentation of host NAD^+^ production? Also, can NR-mitigating effects on an HFD in mice be reproduced in the absence of microbiomes, such as in germfree mice? In a broader context, it is key to point out that in mice, PARP1 knockout (21) and CD38 knockout (22) cause increases in NAD^+^ availability in many tissues and mitigate toxicities caused by an HFD or metabolic stress (21, 22). As these genetically driven changes in NAD^+^ concentrations do not apparently require GI exposures to ingested NAD^+^ precursors such as NR, it begs the question of how much the microbiome is involved in these effects. On the other hand, it is now relevant to ask if the microbiome components are changed in these genetically modified mice and if microbiome effects contribute to the observed phenotypes. Finally, it seems worthwhile to create a healthy dialogue between scientists employing different approaches to physiology studies with NR and NAD^+^ precursors to further advance these studies. In summary, the findings provided by Lozada-Fernández et al. deepen the story of how NAD^+^ precursors can act in mammals and indicate that the field of cellular players is greater than just peripheral, neuroendocrine, or hepatic tissues. The GI tract might be a good place to look as well, if biomedical scientists and clinicians wish to comprehensively understand the effects of 21st-century NAD^+^ precursors as they are translated for use in human medicine.
